# Double Profile Intersection (DoPIo) Ultrasound With Acoustic Radiation Force Tilting Interrogates Young’s Modulus in Transversely Isotropic Media: An In Silico Study

**DOI:** 10.1109/ojuffc.2025.3613275

**Published:** 2025-09-23

**Authors:** SABIQ MUHTADI, KEITA A. YOKOYAMA, CATERINA M. GALLIPPI

**Affiliations:** Lampe Joint Department of Biomedical Engineering, University of North Carolina (UNC) at Chapel Hill, Chapel Hill, NC 27599 USA; North Carolina State University (NCSU), Raleigh, NC 27695 USA

**Keywords:** ARFI, elastography, DoPIo, transversely isotropic (TI) materials, anisotropy, Young’s modulus, shear modulus

## Abstract

This study evaluates the potential for interrogating the Young’s elastic moduli in anisotropic media, including tissue, using Double Profile Intersection (DoPIo) ultrasound. DoPIo is an on-axis acoustic radiation force (ARF)-based elasticity imaging method that quantifies shear elasticity without relying on shear wave propagation. It is hypothesized that by applying a range of ARF excitations that are not perpendicular to the axis of symmetry (AoS) of transversely isotropic (TI) materials and monitoring the resultant variation in DoPIo-measured elasticity versus excitation angle, the Young’s elastic modulus may be interrogated in addition to the shear elastic modulus. The hypothesis was tested *in silico*, and results suggested that while DoPIo outcomes measured at normal (**90**°) ARF-AoS incidence were related to the shear elastic modulus alone, variation in DoPIo-derived elasticity over ARF-AoS incidence angle (defined as **Δ***Elasticity*) exhibited a strong linear correlation with the longitudinal Young’s modulus ($E_{L}$). The results suggest that $E_{L}$ evaluated by the rate of change of **Δ***Elasticity* with ARF-AoS incidence angle may serve as a novel biomarker for characterizing elastically anisotropic tissues such as kidney, skeletal muscle, and breast.

## I. INTRODUCTION

**FIGURE 1.**
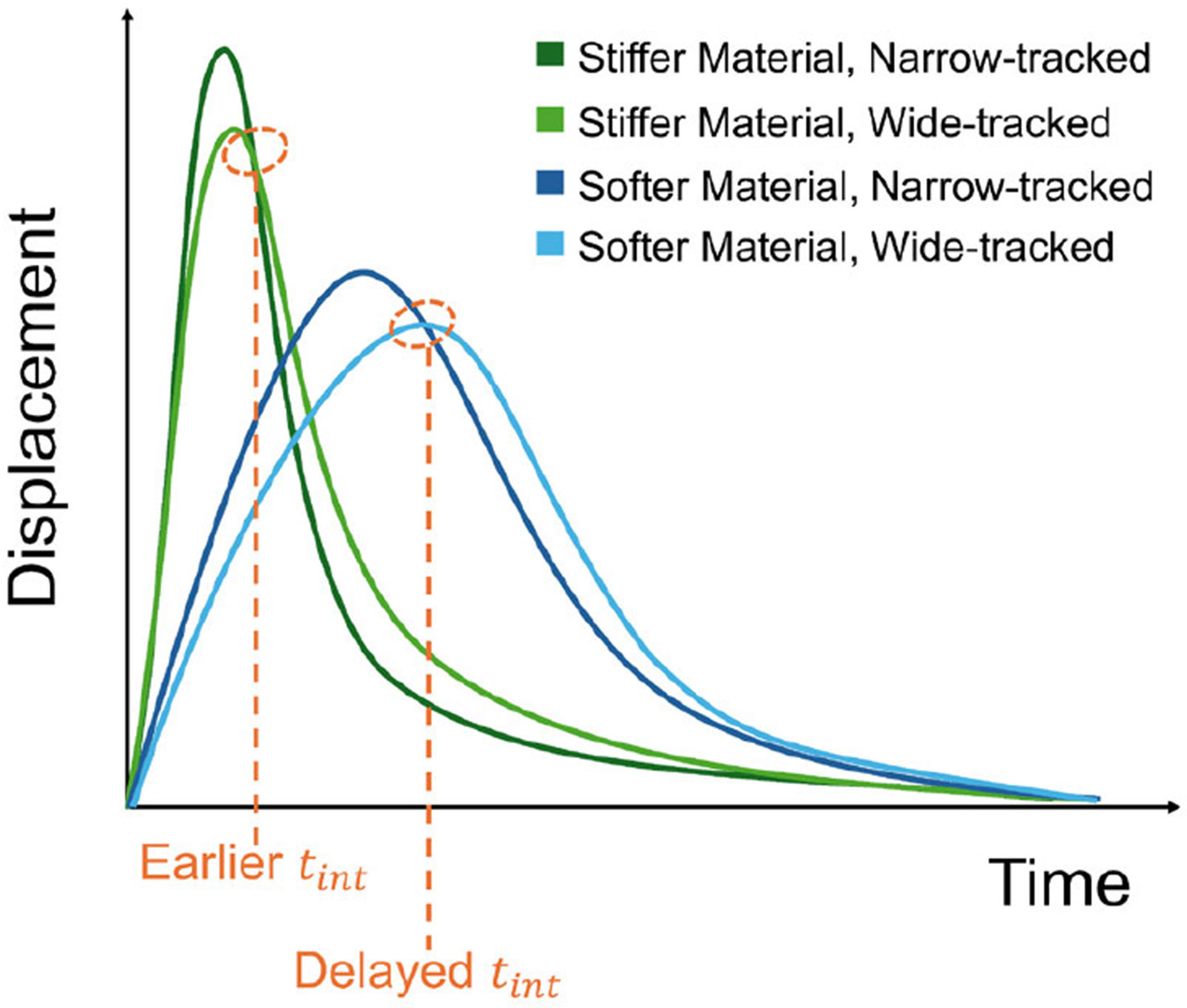
Extraction of $t_{int}$ from DoPIo displacement profiles in stiffer (green) and softer (blue) materials. Darker and lighter shades represent narrow- and wide-tracked profiles, respectively. In the stiffer material, profiles intersect earlier (“Earlier $t_{int}$”), while in the softer material, the intersection is delayed (“Delayed $t_{int}$”).

Ultrasound Elastography (UE) provides a noninvasive means to assess tissue stiffness [1], which is valuable for characterizing soft tissues affected by pathological or physiological changes [2]. Many of these techniques employ acoustic radiation force (ARF) generated by focused ultrasound beams to induce localized mechanical perturbations in tissues, which can then be analyzed to infer tissue mechanical properties [3]. Among these, Double Profile Intersection (DoPIo) ultrasound quantifies shear elasticity of soft tissues by observing displacements on-axis to ARF excitations, without relying on shear wave propagation [4]. In DoPIo, a single ARF excitation induces tissue motion, which is monitored using two different tracking beam focal configurations. One track beam is tightly focused (narrow track) to capture scatterer displacements near the center of the ARF push, while the other beam is more widely focused (wide track) to observe scatterer displacements as they propagate across the width of the tracking point spread function (PSF). The time point at which the resulting two displacement profiles intersect (times-intersect or $t_{int}$) provides insights into the rate of scatterer movement across the tracking PSFs. For example, in stiffer materials, scatterer motion will progress more rapidly across the tracking PSFs, and $t_{int}$ will occur sooner than in softer materials, as demonstrated in Fig. 1. This phenomenon is exploited in DoPIo imaging by relating $t_{int}$ to shear elastic modulus using an empirically derived regression model [4].

The capability of DoPIo to quantify shear elasticity has been demonstrated *in silico* for simulated materials, *in vitro* using calibrated elasticity phantoms, and in excised pig liver, where it produced measurements statistically comparable to shear wave elasticity imaging (SWEI) [4], [5].

Notably, those prior studies evaluated DoPIo in isotropic media; however, most biological tissues - including skeletal muscle, kidney, and breast - are mechanically anisotropic. Such anisotropy can be modelled in the simplest form as transversely isotropic (TI), where the elastic properties differ depending on whether they are measured along or across an axis of symmetry (AoS) [6]. More specifically, the mechanical properties are determined by six independent engineering constants [7]: two Young’s moduli, one aligned along the AoS in the longitudinal (L) direction ($E_{L}$) and the other aligned across the AoS in the transverse (T) direction ($E_{T}$); two shear moduli similarly aligned with respect to the AoS ($\mu_{L}$ and $\mu_{T}$); two Poisson’s ratios, $v_{TT}$ for the plane of isotropy that bisects the AoS and $v_{LT}$ for the plane of symmetry that runs parallel to the AoS; and the constraint,

(1) $$\mu_{T}=\frac{E_{T}}{2(1+v_{TT})}$$

If the TI material is incompressible, the Poisson’s ratios, $v_{TT}$ and $v_{LT}$, have specific values as follows [6], [7]:

(2) $$v_{TT}=1-\frac{E_{T}}{2E_{L}}$$

(3) $$v_{LT}=\frac{1}{2}$$

Prior studies have shown that, when interrogating elasticity in TI materials using ARF-based methods, the orientation of the applied excitation relative to the AoS influences the observed displacements [6], [8], [9], [10]. Specifically, if the ARF excitation is applied at a 90° incidence angle with respect to the AoS, the measured displacements primarily reflect the shear elastic moduli [10]. However, when the incidence angle deviates from 90°, the resulting displacements reflect a combination of both the Young’s and shear elastic moduli [10]. Therefore, we hypothesize that DoPIo performed using normal and non-normal ARF-AoS incidence angles enables the evaluation of both Young’s and shear elastic moduli in TI materials. This hypothesis is herein evaluated *in silico* using DoPIo acquisitions in TI media.

## II. METHODS

### A. IN-SILICO DoPIo SIMULATION FRAMEWORK

Following procedures outlined in [11], ARF push beams were simulated using Field-II [12]. These simulations were based on the specifications of a Philips ATL L7-4 linear array transducer (Philips Healthcare, Bothell, WA) transmitting 300-cycle ARF excitations centered at 4.2 MHz and focused at 25 mm with an F/3.0 focal configuration. Mechanical responses to the ARF excitations were simulated using the FEM solver LS-DYNA3D (Ansys Inc., Canonsburg, PA, USA) in 12 TI materials. The mechanical properties of the TI materials were selected to match those reported for soft tissues such as skeletal muscle, kidney, and breast: $E_{T}=11.74-20.55$ kPa, $E_{L}=35.23-81.58$ kPa, $\mu_{T}=3.20-5.60$ kPa, and $\mu_{L}=4.80-11.20$ kPa [10].

**FIGURE 2.**
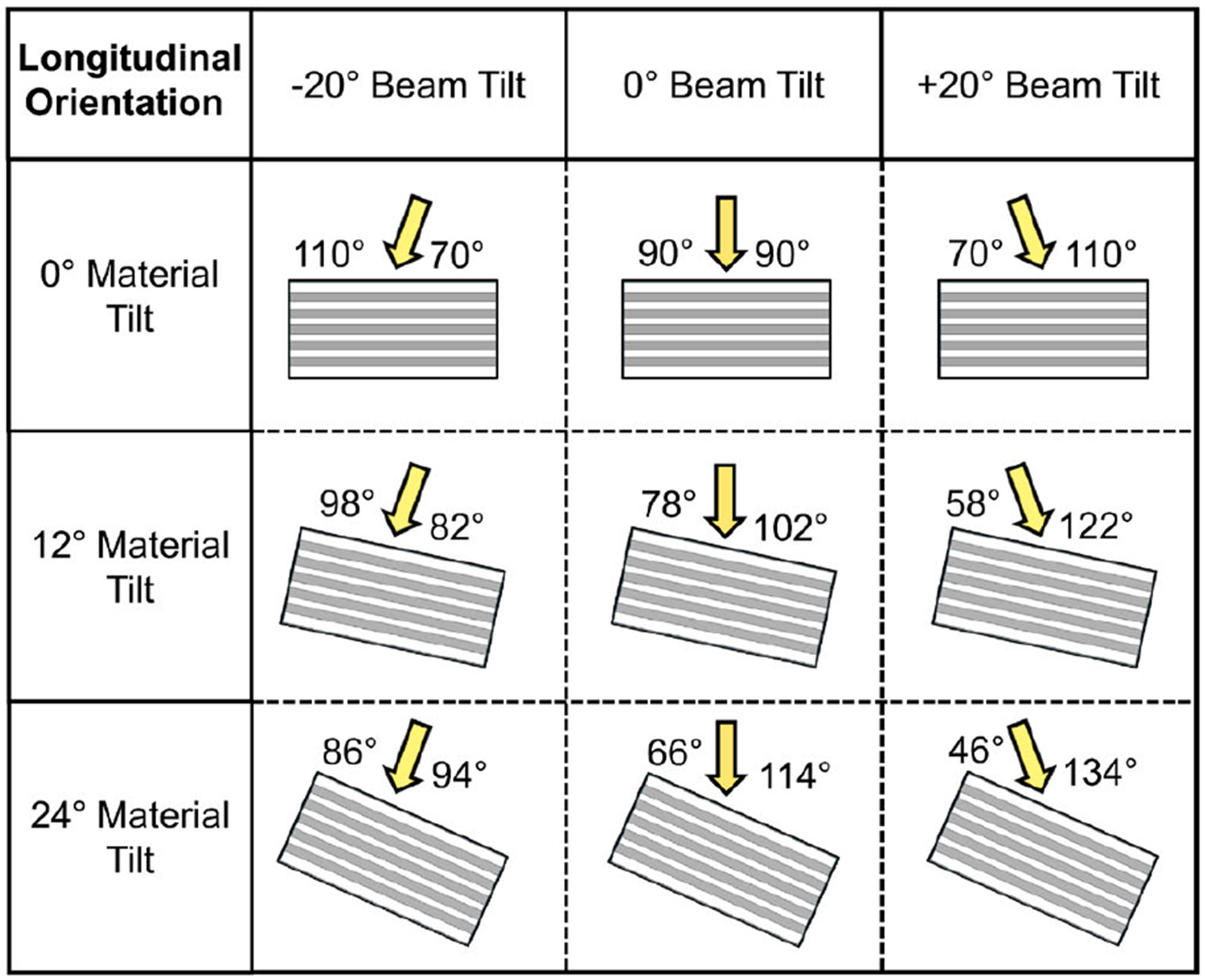
ARF-AoS incidence angles achieved by tilting the ARF push beam (−20°, 0°, 20°) and tilting the TI material (0°, 12°, 24°) while keeping the lateral aspect of the transducer aligned along the material’s AoS (longitudinal orientation).

ARF pushes were applied to each material at various ARF-AoS incidence angles by tilting the simulated push beams by −20° , 0° , and 20°, and by tilting the materials by 0°, 12°, and 24°, as demonstrated in Fig. 2. These variations in ARF-AoS incidence angle were implemented with the transducer aligned along the material’s AoS (longitudinal orientation). Variations with the transducer aligned across the material’s AoS (transverse orientation) were not considered, as elasticity measurements in this orientation are not expected to vary with incidence angle due to the geometry and symmetry of an incompressible TI material [6], [7], [8].

Nodal displacements from the LS-DYNA3D simulations were sampled at 10 kHz, and 10 different realizations of scatterers, with randomized positions and amplitudes, were mapped onto each simulated displacement gradient. For each scatterer realization, displacements were tracked using 2-cycle pulses transmitted centered at 6.2 MHz, focused at 25.0 mm, and simultaneously received using both F/1.5 and F/3.0 focal configurations, with dynamic focusing. These imaging parameters were selected based on prior DoPIo studies [4], [5]. From the received data, two sets of displacement profiles were constructed using 1-D normalized cross-correlation with tracking and search kernel sizes of 2λ and 0.3λ, where λ is the center wavelength [10].

To estimate shear elasticity, the $t_{int}$ between each pair of displacement profiles was extracted and used to compute shear elasticity based on an empirically derived relationship, as detailed in [4] and [5]. Specifically, DoPIo-derived shear elasticity values were evaluated within a ±1*mm* axial range around the focal depth, where the mean and standard deviation were calculated across the 10 scatterer realizations per material.

### B. STATISTICAL ANALYSIS

To evaluate the variation in DoPIo-measured shear elasticity with ARF-AoS incidence angle, the absolute difference in measured elasticity between normal incidence (90°) and all other angles was computed for all materials and defined as Δ*Elasticity*. A linear regression model was then fit to Δ*Elasticity* for each material, and the slope of this relationship was extracted as a summary measure of how DoPIo-derived elasticity changes with ARF-AoS incidence angle.

To determine whether this slope provides insight into the mechanical properties of the TI materials, Spearman’s rank correlation coefficient (*ρ*) was computed between the extracted slope values and the mechanical properties of the materials in longitudinal orientation ($E_{L}$ and $\mu_{L}$). Statistical significance was defined as *p* < 0.05.

## III. RESULTS

**FIGURE 3.**
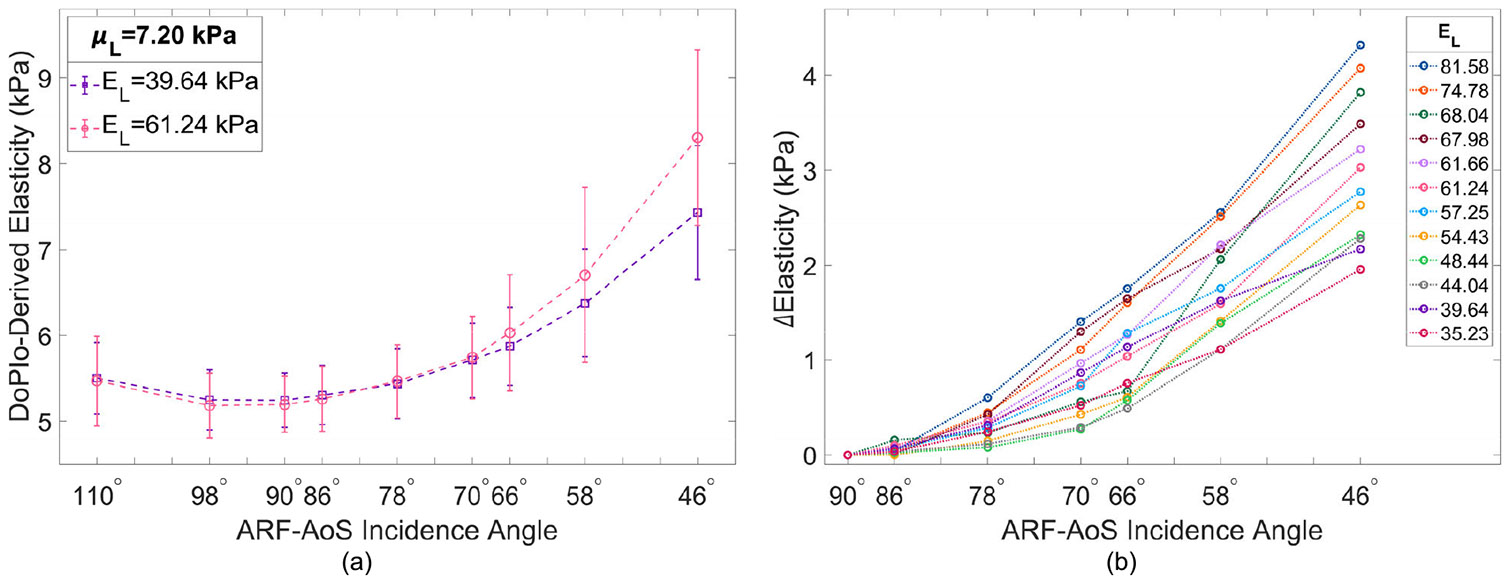
(a) DoPIo-derived elasticity versus ARF-AoS incidence angle in two TI materials with the same $\mu_{L}$ but different $E_{L}$, as specified in the caption; (b)Change in DoPIo-derived elasticity between 90° and all other ARF-AoS incidence angles (Δ*Elasticity*) for all 12 simulated materials in longitudinal orientation. ARF = acoustic radiation force, AoS = axis of symmetry.

Fig. 3(a) illustrates DoPIo-derived elasticity versus ARF-AoS incidence angle for two materials with the same $\mu_{L}$ but different $E_{L}$. Fig. 3(b) illustrates Δ*Elasticity* versus ARF-AoS incidence angle for all materials with respect to $E_{L}$. Table 1 outlines Spearman correlation coefficients and resulting *p*-values between the extracted slope values of Δ*Elasticity* and $E_{L}$ and $\mu_{L}$ of the 12 simulated TI materials.

## IV. DISCUSSION

This study investigated DoPIo’s potential for assessing the Young’s modulus of TI materials, along with its established use for shear modulus estimation, using non-normal ARF-AoS excitation angles. Fig. 3(a) shows DoPIo-derived elasticity as a function of ARF-AoS incidence angle for two TI materials with the same $\mu_{L}$ but different $E_{L}$. At or near 90° incidence, the measured elasticity values are nearly identical, indicating that DoPIo was primarily interrogating $\mu_{L}$. However, as the incidence angle deviated from normal, DoPIo elasticity estimates increased, with larger increases observed in the material with higher $E_{L}$. This suggests that $E_{L}$ influences the degree of change in DoPIo-derived elasticity with ARF-AoS angle. To quantify this relationship, Fig. 3(b) plots the absolute difference in measured elasticity between 90° and all other angles (Δ*Elasticity*) for all 12 simulated materials, annotated by each material’s $E_{L}$. Materials with higher $E_{L}$ exhibited greater Δ*Elasticity* across ARF-AoS angles, resulting in steeper linear regression slopes. A strong correlation was observed between $E_{L}$ and the slope of Δ*Elasticity* (*ρ* = 0.937, *p* < 0.0001), whereas a weaker, non-significant trend was found for $\mu_{L}$ (*ρ* = 0.417, *p* = 0.177). These results highlight $E_{L}$ as the primary contributor to angular variation in DoPIo-derived elasticity in longitudinal orientation and support the potential of DoPIo for characterizing $E_{L}$ in anisotropic tissues such as skeletal muscle, kidney, and breast.

While the mechanical properties of the TI materials evaluated in this study were selected to reflect values reported for soft tissues in literature, the range of $E_{L}$ (35.23–81.58 kPa) was broader than that of $\mu_{L}$ (4.80–11.20 kPa). Although all parameters were co-varied to ensure physically valid TI models, the relatively lower magnitude and narrower variation of $\mu_{L}$ may have contributed to the weaker correlation observed between $\mu_{L}$ and Δ*Elasticity*. Future work will implement materials with a wider range of $\mu_{L}$ values. Additionally, future studies will investigate the clinical relevance of this approach for noninvasive characterization of anisotropic tissue mechanics.

Fig. 3(a) also depicts DoPIo’s performance in evaluating two TI materials with identical longitudinal shear modulus, $\mu_{L}$. At 90° ARF-AoS incidence, it is expected that DoPIo will primarily measure $\mu_{L}$ in the longitudinal orientation and $\mu_{T}$ in the transverse orientation. However, the results show that in the longitudinal orientation, DoPIo underestimates $\mu_{L}$ (actual value = 7.20 kPa, measured value ≈ 5.3 kPa), while in the transverse orientation it overestimates $\mu_{T}$ (actual value = 3.60 kPa, measured value ≈ 4.4 kPa, figure not shown for brevity). This suggests that in both orientations, the measured elasticity is being influenced by a combination of both shear moduli, indicating that DoPIo is unable to fully isolate them. One possible explanation for this limitation is the empirical model currently used for DoPIo measurements, which was developed for isotropic media where tissue response to ARF excitation is governed by a single shear modulus. In TI media, however, both $\mu_{L}$ and $\mu_{T}$ contribute to the tissue’s response, and the existing model does not account for this complexity, leading to underestimation in the longitudinal orientation and overestimation in the transverse orientation. Another contributing factor may be the selected focal configurations of the ARF push and tracking beams, as well as their interrelationship. The width of the push beam affects the amplitude and spatial distribution of induced displacements, while the tracking beam widths determine the span of displacements captured for estimating $t_{int}$. Both factors directly impact $t_{int}$ measurements and thus influence DoPIo’s sensitivity to shear elasticity and elastic anisotropy. This study employed an F/3.0 push in combination with F/1.5 and F/3.0 tracking beams, consistent with prior DoPIo studies that identified this configuration as optimal for isotropic materials [5]. However, the influence of push-and-track focal geometry on DoPIo measurements in TI media has not been fully characterized and will be the focus of future investigation.

Despite these limitations, the long-term goal of using DoPIo for full characterization of incompressible TI materials remains feasible. In principle, three independent elastic constants—$\mu_{L}$, $\mu_{T}$, and $E_{L}$—must be measured, with the remaining parameters ($E_{T}$, $v_{TT}$, and $v_{LT}$) determined via established constitutive relationships. Under ideal conditions, DoPIo measurements at 90° ARF-AoS incidence in the longitudinal orientation would estimate $\mu_{L}$, while measurements in the transverse orientation would estimate $\mu_{T}$. As demonstrated in this study, Δ*Elasticity* correlates strongly with $E_{L}$, suggesting that $E_{L}$ may be interrogated through angular sensitivity. Together, these three measurements would enable full reconstruction of TI elasticity. However, the observed under- and overestimation of $\mu_{L}$ and $\mu_{T}$, respectively, indicate that improvements to the underlying DoPIo model are necessary before this ideal framework can be reliably implemented. Future work will focus on model refinement to enable more accurate and decoupled estimation of $\mu_{L}$ and $\mu_{T}$, thereby advancing DoPIo toward full quantitative characterization of TI soft tissues.

DoPIo-derived measurements at ARF-AoS incidence angles corresponding to material tilts of −12° and −24° were not included in this study. This decision was based on the assumed symmetry of TI materials about their AoS, which implies that positive and negative material tilts of equal magnitude produce equivalent mechanical responses. Therefore, only positive material tilt angles (0° , 12° , and 24°) were analyzed in this study to avoid redundancy.

The variations in ARF-AoS excitation angles evaluated in this study were achieved using a combination of beam tilting and material tilting. While electronic beam steering would be more practical for future *ex vivo* and *in vivo* studies, it has the potential to introduce distortions to the transmitted beam shape, focal position, and side lobe prominence, which will need to be addressed in future investigations.

A key limitation of this simulation-based study is the assumption of a constant ARF body force across all materials. In theory, if the medium is linearly elastic, variations in ARF magnitude should not affect the measured DoPIo $t_{int}$, as displacement profiles would scale proportionally while their intersection time remains unchanged. However, in practical *in vivo* or *in vitro* settings, this assumption may not hold due to additional sources of variability. Patient-specific factors such as BMI, motion, tissue attenuation, and acoustic path length can influence the delivered force profile and, more importantly, may distort the shape of both the ARF excitation and tracking beams. These distortions can affect the spatial characteristics of the displacement fields and alter the resulting $t_{int}$ measurements. While the influence of ARF magnitude in linearly elastic media is under ongoing investigation, addressing beam distortion effects will be critical for translating DoPIo to experimental and clinical use. Future work will explore strategies to compensate for these factors, such as using reference materials, implementing beam-shape correction models, or developing calibration techniques to account for inter-subject variability and improve robustness across imaging scenarios.

Another limitation of this study is that the *in silico* results were derived from simulations of only 12 TI materials. While these materials represent a wide range of elasticities typical of various soft tissues, including skeletal muscle, kidney, and breast, future studies involving a larger number of materials could help refine the optimal ARF-AoS excitation angle range and step size, as well as assess the feasibility of electronic beam steering in clinical applications.

This study complements our prior work using Viscoelastic Response (VisR) ultrasound to assess TI media [10], but differs substantially in both method and interpretation. VisR provides qualitative measures of tissue stiffness, and our previous study showed that the percentage change in VisR-derived elasticity across ARF-AoS angles correlated with the $\mu_{L}∕E_{L}$ ratio. In contrast, DoPIo is a quantitative technique designed to estimate shear elasticity. Although we observed that DoPIo underestimates $\mu_{L}$ in longitudinal orientation and overestimates $\mu_{T}$ in transverse orientation at 90° incidence, the angular dependence of DoPIo-derived elasticity was found to correlate strongly with $E_{L}$ alone. This suggests that, despite model limitations in isolating individual shear moduli, DoPIo captures stiffness variations directly linked to Young’s modulus in TI tissues—an insight not accessible with VisR.

## V. CONCLUSION

This study has shown that DoPIo ultrasound can be used to interrogate Young’s and shear elastic moduli in TI materials. *In silico*experiments revealed that in longitudinal orientation, with the transducer aligned along the material’s axis of symmetry (AoS), the rate of change in DoPIo elasticity measurements at non-normal ARF-AoS excitation angles with respect to measurements taken at 90° incidence correlated strongly with the longitudinal Young’s ($E_{L}$) elastic modulus of the materials. Evaluated in this manner, $E_{L}$ has the potential to serve as a novel semi-quantitative biomarker for interrogating the mechanical properties of anisotropic tissues such as kidney, skeletal muscle, and breast.

## Figures and Tables

**TABLE 1.** Spearman’s correlation coefficient (*ρ*) and corresponding *p*-values between Δ*Elasticity* slope and $E_{L}$, $\mu_{L}$.

Factor	Correlation	*p*-value
Slope vs. $E_{L}$	0.937	<0.0001
Slope vs. $\mu_{L}$	0.417	0.177
